# Genetic and phenotypic diversity of microsymbionts nodulating promiscuous soybeans from different agro-climatic conditions

**DOI:** 10.1186/s43141-022-00386-5

**Published:** 2022-07-18

**Authors:** Simon W. Mburu, Gilbert Koskey, Ezekiel M. Njeru, Omwoyo Ombori, John Maingi, Jacinta M. Kimiti

**Affiliations:** 1 Department of Biochemistry, Microbiology and Biotechnology, Kenyatta University, P.O. Box 43844 (00100), Nairobi, Kenya; 2 Department of Biological Sciences, Chuka University, P.O Box 109, Chuka, –60400 Kenya; 3 Institute of Life Sciences, Scuola Superiore Sant’Anna, Piazza Martiri Della Libertà, 33, 56127 Pisa, Italy; 4 Department of Plant Sciences, Kenyatta University, P.O. Box 43844 (00100), Nairobi, Kenya; 5 Department of Forestry and Land Resources Management, South Eastern Kenya University, P.O. Box 170, Kitui, -90200 Kenya

**Keywords:** Principal coordinate analysis, Shannon’s information index, Bradyrhizobia, 16S rRNA gene, ARDRA

## Abstract

**Background:**

Global food supply is highly dependent on field crop production that is currently severely threatened by changing climate, poor soil quality, abiotic, and biotic stresses. For instance, one of the major challenges to sustainable crop production in most developing countries is limited nitrogen in the soil. Symbiotic nitrogen fixation of legumes such as soybean (*Glycine max* (L.) Merril) with rhizobia plays a crucial role in supplying nitrogen sufficient to maintain good crop productivity. Characterization of indigenous bradyrhizobia is a prerequisite in the selection and development of effective bioinoculants. In view of this, bradyrhizobia were isolated from soybean nodules in four agro-climatic zones of eastern Kenya (Embu Upper Midland Zone, Embu Lower Midland Zone, Tharaka Upper Midland Zone, and Tharaka Lower Midland Zone) using two soybean varieties (SB8 and SB126). The isolates were characterized using biochemical, morphological, and genotypic approaches. DNA fingerprinting was carried out using 16S rRNA gene and restricted by enzymes *Hae*III, *Msp*1, and *EcoR*I.

**Results:**

Thirty-eight (38) bradyrhizobia isolates obtained from the trapping experiments were placed into nine groups based on their morphological and biochemical characteristics. Most (77%) of the isolates had characteristics of fast-grower bradyrhizobia while 23% were slow-growers. Restriction digest revealed significant (*p* < 0.015) variation within populations and not among the agro-climatic zones based on analysis of molecular variance. Principal coordinate analysis demonstrated sympatric speciation of indigenous bradyrhizobia isolates. Embu Upper Midland Zone bradyrhizobia isolates had the highest polymorphic loci (80%) and highest genetic diversity estimates (*H’* = 0.419) compared to other agro-climatic zones.

**Conclusion:**

The high diversity of bradyrhizobia isolates depicts a valuable genetic resource for selecting more effective and competitive strains to improve promiscuous soybean production at a low cost through biological nitrogen fixation.

## Background

Low-cost agricultural technologies are gaining more attention in the global pursuit of sustainable food production models. For instance, there is growing demand to double food production in Africa to feed the anticipated 2.5 billion people by 2050 [36]. Soybean (*Glycine max* [L.] Merrill) is a grain legume that is rich in carbohydrates, oil, proteins, vitamins (vitamins E, K, niacin, thiamine, and choline), digestible fiber, and minerals such as Mg, Zn, K, Cu, and Fe [29]. Globally, soybean production accounts for about 68% of the total legume production and about 80% of the land area dedicated to legume cultivation. Kenya produces about 5000 metric tons of soybean annually while the demand is estimated at 100,000 to 200,000 metric tons per year, requiring importation to fulfill the domestic demand of approximately 95% [9]. Soybean production is constrained by several factors, including high cost of inputs and lack of compatible microsymbionts in the soils. Promiscuous soybean varieties have been bred to overcome the problem of nodulation by forming symbiotic associations with diverse indigenous soil bradyrhizobia [27].

There are diverse nitrogen-fixing bacteria that nodulate with soybean and the majority belong to *Mesorhizobium*, *Bradyrhizobium*, and *Sinorhizobium* genera [25, 57]. The strains are either slow growers or fast growers. In sub-Saharan Africa, the most common and widely distributed strains are the slow growers which comprise of *Bradyrhizobium yuanmingense* [54], *Bradyrhizobium japonicum*, *Bradyrhizobium liaoningense*, and *Bradyrhizobium elkanii* [22, 58]. Fast growers consist of *Sinorhizobium xinjiangense*, *Sinorhizobium fredii* [16], and *Mesorhizobium tianshanese* [56]. According to Naamala et al. [30], *B. elkanii* and *B. japonicum* are common worldwide, inhabiting different climatic conditions. Kenya has a warm tropical climate that favors *B. japonicum* and *B. yuanmingense.* Studies from already characterized *Bradyrhizobium* strains have shown that promiscuous soybeans can nodulate effectively with genetically distinct species [3]. The ability of bradyrhizobia strains to establish a symbiotic association that facilitates nitrogen acquisition and utilization in soybeans [44], obviates the need for external supply of nitrogen through microbial inoculation or inorganic fertilizers [6, 11]. Additionally, bradyrhizobia and other plant growth-promoting rhizobacteria (PGPR) have the potential to offer sustainable alternative solutions to problems related with stress tolerance and crop yield [2].

Field trials have proven that the population of indigenous bradyrhizobia strains compatible with promiscuous soybeans are occasionally available in the soil, and if present, they are either infective in fields where soybeans have not been previously grown [41]. In this view, Gyogluu et al. [13] demonstrated the need to assess the compatibility of various promiscuous soybean genotypes to a local bradyrhizobia strain and enhance plant growth and yields in poor agricultural fields in Mozambique. This study, however, used a single strain and did not consider the selection of different native bradyrhizobia strains that could potentially elicit better symbiotic performance with the tested soybean genotypes. Nonetheless, other studies have shown that despite the successes in the development of promiscuous soybeans that nodulate with diverse bradyrhizobia strains, their yields are still meager [47]. This necessitates establishing a local diversity of indigenous bradyrhizobia strains adaptable to various environmental stresses and compatible with promiscuous soybean cultivars in enhancing plant growth and productivity [32, 45].

The application of commercial inoculants on promiscuous soybeans to improve yield has failed due to the inadaptability of the introduced strains to the local climatic and soil conditions [52]. Most of the commercial inoculants used in Africa for soybean production originate from the USA and Asia [1]. The inoculum efficiency is usually poor, presumably due to their inability to establish effective symbiosis with the African-bred soybean genotypes [38]. Besides, promiscuous soybean cultivation history in Eastern Kenya is relatively brief and no record of inoculation despite the high potential [33]. Besides, no study has been carried out on the range of genetic and morphological diversity of indigenous bradyrhizobia from agricultural farms in Eastern Kenya. Therefore, the determination of genetic diversity of the promiscuous soybean-nodulating bradyrhizobia, within a local geographical scale, can promote the adaptability and resilience of the inoculants to prevailing climatic and soil conditions. Gyogluu et al. [13] also suggest that the diverse genetic pool of bradyrhizobia enhances the stability of promiscuous soybeans and increases adaptability in different climatic conditions. The objectives of this study were to isolate indigenous bradyrhizobia isolates that nodulate with promiscuous soybeans and to determine their genetic diversity in four contrasting agro-climatic conditions of Eastern Kenya.

## Methods

### Study sites

The bradyrhizobia isolates were trapped in the smallholder farmers’ fields in Embu and Tharaka-Nithi Counties of Eastern Kenya. The study sites in Embu County were located at 0.53° S, 37.45° E on the slopes of Mt. Kenya. The rainfall is bimodally distributed, with short rains occurs from October to December while long rains are from March to July. Precipitation annually range from 800 to 1200 mm while climate is humid to semi-humid. The region is characterized by high population density and small farm-size which are fragmented. The land-use system comprises maize, common beans, soybeans, sorghum, cowpea, nuts, avocado, pawpaw, bananas, and cash crops such as tea and coffee. The farms have no history of bradyrhizobia inoculation, but production depends on the quantity of inorganic fertilizers commonly applied. The soils in the region are characterized as deep humic top-soil, brown to dusky red, clay to clay roam, and well-drained. Soil structure is weak due to high cultivation frequency and appears dusty in dry seasons. In Embu County, the two agro-climatic zones were Embu Upper Midland Zone (EUMZ) (1500–2000 m asl) located at 0° 22’ 41.3’’ S, 37° 33’ 29.7’’ E and Embu Lower Midland zone (ELMZ) (1000–1500 m asl) located at 0° 25’ 05.2’’ S, 37° 37’ 14.6’’ E.

Tharaka-Nithi County lies on the slopes of Mt. Kenya on the Eastern side. The area experiences an average maximum rainfall of 1200 mm and a minimum of 400 mm annually whose distribution pattern is bimodal. The short rains occur in October to December while long rains take place in March to July. The dominant crops are maize, soybeans, common beans, peas, millet, sweet potatoes, sugarcane, avocado, bananas, coffee, cassava, and guavas [28]. The farms are characterized by extensive application of inorganic fertilizers and no record of rhizobia inoculation. The soils in lower midland zones are acidic and range from shallow to deep red clay soil. The upper midland zone towards the basement of Mt. Kenya soils are shallow and rocky although others are shallow with loam and well-drained. In Tharaka-Nithi County, the isolates were trapped in two agro-climatic zones, Tharaka-Nithi Upper Midland Zone (TUMZ) (1500–2000 m asl) at 0° 22’ 16.8’’ S, 37° 35’ 17.8’’ E and Tharaka-Nithi Lower Midland zone (TLMZ) (1000–1500 m asl) located at 0° 23’ 02.5’’ S, 37° 40’ 13.8’’ E.

### Soil sampling and analysis

Before the rains, soil sampling was carried out randomly in 20 points in every selected study site in each agro-climatic zone. Soil samples were collected in the upper part (5–20 cm) of the soil horizon and mixed thoroughly to make a homogenous composite sample. A sub-sample (1 kg) of the composite samples per site was packed independently to avoid cross-contamination for analysis as described by Somasegaran and Hoben [49]. The samples were oven-dried at 60℃, mixed homogenously, and then sieved (sieve size 2 mm) before subjected to physical–chemical analysis. The soil pH was established at a ratio of 1:2, soil to water suspension as described by Rayment and Higginson [37]. Mehlich-3 method was used to determine potassium (K) and phosphorus (P) [48]. Walkley–Black oxidation method was used to determine soil organic carbon, while the hydrometer method was used to determine the size of soil particles [31]. The total nitrogen was determined using the Kjeldahl method [5].

### Field trapping and morphological characterization of bradyrhizobia isolates

Nodules were collected from two promiscuous soybean varieties (SB8 and SB126) grown in smallholder farmers’ fields located in four different agro-climatic zones of eastern Kenya. The SB8 and SB126 soybean varieties used as test plants were obtained from the Kenya Seed Company, Nairobi. The two varieties are high-yielding and suitable for African tropics. They were developed by the International Institute of Tropical Agriculture (IITA) to nodulate and fix nitrogen with diverse indigenous bradyrhizobia strains present in the soil. The varieties have indeterminate growth characterized by continued vegetative activity throughout the flowering period. They form unifoliate primary leaves that are ovate and opposite, while secondary leaves are trifoliate and alternate. SB8 variety has an early maturity compared to SB126 variety that matures late [51]. Before the onset of the short rains (March to August), the farms were ploughed and harrowed, then demarcated in to small plots (3 m × 3 m size). Soybean seeds of uniform size, shape, and color were planted in furrows with a spacing of 45 cm by 60 cm. A randomized complete block design (RCBD) was used in distributing the treatments, and each treatment was replicated three times. To supplement the phosphate content in the soil, triple super-phosphate (45% P_2_O_5_) was added. Nodule sampling was carried out at the flowering stage where in every site, five plants were randomly sampled, and 5–10 nodules were excised from each plant. The selected nodules were dark-red in color which is an indication of the presence of the leghemoglobin that actively takes part in nitrogen-fixing activity.

The nodules were surface sterilized using 3.5% NaOCl for 3 min, then rinsed with sterile distilled water six times to remove NaOCl traces [53]. Clean nodules were crushed separately after sterilization using a sterile glass rod, and a loopful of the suspension was inoculated on yeast extract mannitol agar (YEMA) plates and incubated in the dark at 28 °C for 48 to 72 h as described by Somasegaran and Hoben [49]. Re-inoculation of single colonies was done using the same media until pure single colonies of bradyrhizobia were obtained. The isolates and the two reference strains (USDA110 and USDA136 obtained from Mea Company Limited Kenya) were streaked on YEMA media supplemented with bromothymol blue and Congo red dyes to confirm the purity. After purification, the isolates were stored for a short-term on YEMA slants maintained at 4 °C while for long-term storage, cultures were maintained in YEMA broth with glycerol (30%) at − 80 °C. The bradyrhizobia isolates were characterized for morphological and biochemical characteristics.

### Authentication of bradyrhizobia isolates

Authentication and symbiotic effectiveness of representative bradyrhizobia isolates based on morphological and biochemical characteristics was carried out to confirm their ability to re-nodulate promiscuous soybean plants in the greenhouse under biologically controlled conditions in a complete randomized design (CRD) [49]. Seeds of uniform size for two soybean varieties SB8 and SB126 were surface sterilized using 3.5% NaOCl for 5 min and washed thoroughly with five changes of sterile distilled water, and two seeds were planted in each Leonard jar assembly. After germination, the seedlings were thinned to one plant per Leonard jar assembly.

The soybean seedlings were inoculated with 1 mL (∼10^9^ cells mL^−1^) of bradyrhizobia isolates cultured for 4 days incubated at 28 °C in YEMA broth as described by Somasegaran and Hoben [49]. The treatments comprised of indigenous bradyrhizobia isolates, commercial inoculant (Biofix), and a negative control deprived of rhizobia inoculation. The experiment was arranged in a completely randomized design with four replicates. The plants were supplied weekly with nitrogen-free growth solution [7]. For the positive nitrogen control treatment, 0.05% KNO_3_ was added weekly to give a nitrogen concentration of 70 g kg^−1^. Negative control, which comprised of non-inoculated N-free plants, together with rhizobia treatments were supplied with sterile distilled water weekly. The pH of the solution was adjusted to 6.8 using 1.0 N NaOH or 1.0 N HCl. After 45 days, soybean plants were carefully uprooted from the planting medium, and the ability (presence or absence) of the isolates to induce nodulation was assessed and quantified for authentication. Nodule number, nodule appearance, and nodule dry weight were also documented. Plant shoots were oven dried for 72 h at 70 °C before weighing the shoot dry weight. Symbiotic effectiveness was assessed using the following formular.

$$\mathrm{SE}=\frac{\mathrm{SDW of inoculated soybean plants}}{\mathrm{SDW of uninoculated soybean plants treated with nitrogen }\left({0.05\mathrm{ KNO}}_{3}\right)}\mathrm{ X} 100$$

**SDW** = Shoot dry weight.

**SE** = Symbiotic efficiency.

### Genetic diversity of bradyrhizobia isolates

#### DNA extraction and PCR amplification of 16S rRNA gene

The bradyrhizobia isolates (38 isolates) and the two reference strains prior to DNA extraction were cultured on YEMA for 4 days at 28ºC. The DNA was extracted using Quick-gDNA™ MiniPrep DNA extraction kit (Zymo Research, UK) as described by the manufacturer. The resulting DNA pellet was used to run PCR. In each PCR reaction, the total volume of 25 μl contained 2.5 μl of 10 × PCR buffer, 0.5 μl dNTPs, 0.7 μl of Taq DNA polymerase, 0.4 μl of 10 μM primer Y1, 0.4 μl of 10 μM primer Y3, 1.5 μl of 1 mmol L^–1^ MgCl_2_, and 15 μl deionized water (BioLab). The DNA template (4 μl) was then added to the PCR reaction mix. The sequence of the forward primer Y1 (5’-TGGCTCAGAACGAACGCTGGCGGC-3’) corresponding to positions 20–43 while the reverse primer Y3 (5’-TACCTTGTTACGACTTCACCCCAGTC-3’) corresponding to positions 1482–1507 for 16S rDNA sequence of *Escherichia coli* were used. The PCR amplification conditions comprised of initial denaturation for 2 min at 95ºC, followed by 37 cycles (denaturation at 94ºC for 40 s, annealing at 55ºC for 30 s, extension at 94ºC for 2 min), and final extension at 72ºC for 10 min. The PCR products were stored at 4ºC. During the PCR experiment, three biological replicates and three technical replicates were used to ensure reproducibility of results and consistency of the measuring tool, respectively.

The PCR products (5 μl) were stained using SYBR green (BioLab) and separated in agarose (1.4% w/v) gel electrophoresis in TBE buffer. Electrophoresis was run at 80 V for 50 min. A molecular marker (1 kb plus DNA ladder) was used to estimate the molecular size of the bands. The bands were visualized under a UV trans-illuminator lamp after gel electrophoresis and photographed.

#### 16S rRNA gene restriction fragment analysis

Restriction fragment analysis of the PCR products (5 μl each) was carried out for all the isolates. Restriction enzymes *Hae*III, *Msp*I, and *EcoR*I (BioLab), 10 μl each was used separately. The digested fragments were stained using SYBR green (0.4 μl) and separated at 80 V for 50 min in agarose (1.4% w/v) gel electrophoresis in tris–acetate-EDTA (0.5XTAE) buffer. Fragment sizes were estimated using a 100-kb DNA ladder. The UV trans-illuminator lamp was used to visualize restriction products.

### Statistical analyses

The SAS statistical package version 9.0 was used for analysis of molecular variance, and means were compared using Tukey’s HSD post hoc test at *p* < 0.05 level of significance. Amplified Ribosomal DNA Restriction Analysis (ARDRA) was used to determine the genetic diversity of bradyrhizobia isolates using Jaccard similarity in Gene Alex software (version 6.5). The restriction fragments from the three enzymes were scored as absent (0) and present (1), and only reproducible bands were considered. The restriction patterns from the three enzymes were combined, and maximum likelihood method was used to cluster similarity matrix into dendrogram using DARwin software (version 6.0.021). Analysis of molecular variance (AMOVA), Nei unbiased genetic distance, percentage of polymorphic loci (% P), and effective number alleles (Ne) were used to test the genetic structure of bradyrhizobia isolates among the soybean host plants and between the four agro-climatic zones using Gene Alex software (version 6.5). Canonical correspondence analysis (CCA) was used to analyze correlation between environmental factors and microbial diversity in different agroecological zones using CANOCO windows software version 4.5 (Biometrics, Netherlands). The relationships between agro-climatic zones and bradyrhizobia isolates was determined by performing principal component analysis (PCA) in the PAST program (version 4.03). Shannon-Weiner diversity index (*H*’) was used to assess diversity and evenness based on 38 bradyrhizobia isolates [24]. The equation used for the diversity index was:

$${H}^{^{\prime}}=-\sum (Pi \mathbf{I}\mathbf{n} Pi)$$

where *Pi* was expressed as *ni/N*, which was the dominance of the bradyrhizobia isolates, *ni* referred to the number of bradyrhizobia isolates per cluster at a given site, and *N* was the total number of bradyrhizobia isolates in a site. 

## Results

### Soil properties

Most of the soils from the study sites were characteristically acidic, where TUMZ had the lowest pH (4.52) compared to other agro-climatic zones (Table 1). TLMZ and ELMZ soil also had low pH of 5.42 and 5.01, respectively, while soil sampled from EUMZ was slightly acidic with a pH of 6.31. Available phosphorus ranged from 17.51 g kg^−1^ in soils obtained from ELMZ to 27.00 g kg^−1^ in soils from EUMZ. The soils from TUMZ and TLMZ had available P of 26.50 g kg^−1^ and 21.00 g kg^−1^, respectively (Table 1).

**Table 1:** Soil properties from the experimental sites

Properties	TUMZ	TLMZ	EUMZ	ELMZ
pH	4.52	5.42	6.31	5.01
OC (%)	2.98	2.49	3.29	3.06
N (%)	0.26	0.42	0.25	0.28
K cmol kg^−1^	0.70	1.50	1.00	0.40
P (g kg^−1^)	26.50	21.00	27.00	17.51
Sand (%)	51	57	45	47
Clay (%)	35	21	53	41
Silt (%)	14	22	39	12
Texture class	Sandy clay	Sandy clay loam	Clay	Sandy clay
Isolates	TU1-TU7	TL1-TL13	EU1-EU13	EL1-EL5
Isolates % distribution	18	34	34	13

*OC* Organic carbon, *K* Potassium, *N* Nitrogen, *ELMZ* Embu Lower Midland Zone, *EUMZ* Embu Upper Midland Zone, *TLMZ* Tharaka-Nithi Lower Midland Zone, *TUMZ* Tharaka-Nithi Upper Midland Zone

The soil % N ranged from 0.25% in soils from EUMZ to 0.42% in soils from TLMZ (Table 1). The percentage organic carbon from the soils ranged from 2.49% in TLMZ to 3.29% in EUMZ. The concentrations of exchangeable potassium ions (K^+^) in soils ranged from 0.40 cmoL kg^−1^ in soils from ELMZ to 1.50 cmoL kg^−1^ in soils from TLMZ. The clay content in the soils ranged from 21 to 53%, and the soil from EUMZ had the highest clay content. The soil texture from the farms was either clay, sandy clay loam, or sandy clay (Table 1).

### Isolation and authentication of bradyrhizobia isolates

During the study, a total of 38 pure bradyrhizobia isolates were obtained from the root nodules of two soybean varieties grown during field trapping experiments in Embu and Tharaka-Nithi Counties. Tharaka-Nithi Lower Midland Zone (TLMZ) and Embu Upper Midland Zone (EUMZ) had the highest number of isolates, recording 13 isolates each. In comparison, Tharaka-Nithi Upper Midland Zone (TUMZ) and ELMZ had 7 and 5 isolates, respectively. Soybean SB126 had the highest number of isolates (22 isolates) while SB8 had 16 isolates. All the bradyrhizobia isolates were able to re-nodulate the two promiscuous soybean varieties used during the authentication experiment in the greenhouse. There was significant variation in nodulation level from one isolate to another. Commercial inoculant (Biofix) also nodulated effectively with the two soybean varieties while in non-inoculated treatment, no nodules were observed. These findings confirms that the 38 bacterial isolates were bradyrhizobia due to their ability to re-nodulate the soybean varieties. In the greenhouse bioassay, inoculation of soybeans with native bradyrhizobia had significant effect (*p* < 0.001) on nodule and shoot dry weight with bradyrhizobia isolate RI9 recording the highest performance. The best performing isolates (RI9 and RI2) from greenhouse set up outperformed commercial bradyrhizobia (USDA110) in terms of symbiotic effectiveness of 119.17%, 142.35%, and 101.01%, respectively, when compared to nitrogen control (Table 2).

**Table 2:** Shoot dry weight, nodule dry weight, and symbiotic efficiency of two soybean varieties inoculated with bradyrhizobia isolates in the greenhouse

Varieties	SDW (g plant^−1^)	NDW (mg plant^−1^)	SE%
SB8	0.33^a^	5.02^a^	74.06^b^
SB126	0.31a	2.99^ab^	98.70^a^
**Isolates**			
RI3	0.42^abc^	1.43^b^	99.82^ab^
RI4	0.48^ab^	4.85^b^	119.17^b^
RI5	0.32^bcde^	1.16^b^	81.25^bcde^
RI6	0.28^bcde^	0.37^b^	67.36^bcdef^
RI7	0.19^cde^	0.39^b^	39.62^cdef^
RI8	0.35^abcd^	2.21^b^	84.84^abcd^
RI9	0.58^a^	19.28^a^	142.35^a^
ICT	0.33^bcde^	8.73^ab^	84.45^abcd^
BT	0.43^abc^	2.17^b^	101.01^ab^
NT	0.43^abc^	0	100.00^ab^
BICT	0.39^abc^	0.38^b^	94.45^abc^
UT	0.09^e^	0	0
***P*-Values**			
Isolates	0.001	0.004	0.001
Variety	0.475	0.102	0.005
Variety *isolates	0.809	0.866	0.715

Key: *SDW* Shoot dry weight, *NDW* Nodule dry weight, *SE* Symbiotic efficiency, * interaction. Mean followed by different superscript letters ^a-f^ within each column differ significantly at *p* < 0.05. Consortium, BICT consortium + commercial inoculant, BT commercial inoculant, UT uninoculated (− ve treatment), *NT*, nitrogen treatment (+ ve treatment), BICT consortium + commercial inoculant

### Morphological and biochemical characteristics of bradyrhizobia isolates

Most of the isolates (77%) had a characteristic of fast-growing bradyrhizobia since they turned YEMA media supplemented with BTB dye from deep green to yellow (Table 3). The observation indicated the production of acidic substances which diffused into the media. The other isolates turned BTB medium from deep green to blue which is a typical characteristic of slow-growing bradyrhizobia due to the production of alkaline substances in the medium. In addition, all the isolates absorbed little or no Congo red when incubated in the dark. The isolates, as revealed by Gram’s staining reaction, were Gram-negative and rod-shaped. The isolates had larger colonies with a diameter ranging between 0.5 and 5 mm, and they showed mucus (exo-polysaccharide) production on the growth medium. The isolates had different colors which were cream white, white, and milky white. Most (77%) isolates were translucent on transparency while 23% were opaque. The elevation of the isolates colonies was either raised, domed, or convex. The 38 isolates obtained were grouped into nine morphotypes based on their morphological characteristics. The most abundant was morphotype IX, accounting for 40% of the total isolates followed by morphotype IV with 22%. Morphotypes III and VI were the least abundant isolates detected with only 3% each (Table 3). The reference strain USDA 110 grouped with most isolates in morphotype IX while USDA 136 was in morphotype VII. The morphological results of these isolates closely matched those of bradyrhizobia as described by Somasegaran and Hobed [49] based on the use of selective media (YEMA) that target only rhizobia.

**Table 3:** Morphological and biochemical traits of bradyrhizobia isolates

	Congo Red Absorption	BTB Reaction	Margin	Color	Elevation	Gram Stain	Transparency	Size (mm)	Colony shape	Texture	%	Nodulation
I	Crna	Y	S	Cw	Rs	− ve	O	0.5	C	Sg	5	0
II	Crna	Y	S	Mw	Dmd	− ve	O	1	C	G	4	0
III	Crna	Y	Sc	Ww	Cvx	− ve	T	3.5	C	Sg	3	3.08 ± 0.65c
IV	Crna	Y	Sc	Ww	Rs	− ve	T	5	C	Sg	22	4.00 ± 0.94c
V	Crna	Y	Sc	Ww	Cvx	− ve	T	0.5	C	Sg	5	3.08 ± 1.16c
VI	Crna	B	Sc	W	Rs	− ve	T	1.5	C	Sg	3	1.42 ± 0.26d
VII	Crna	Y	Sc	Mw	Cvx	− ve	T	0.5	C	Fg	13	1.33 ± 0.25d
VIII	Crna	B	Sc	Mw	Cvx	− ve	T	1.5	C	Sg	5	6.91 ± 2.49b
IX	Crna	Y	Sc	Mw	Cvx	− ve	T	4	C	Sg	40	9.25 ± 1.77a

*Crna* Congo red non-absorbing, *Cw* Creamy white, *Y* Yellow, *S* Smooth, *Mw* Milky white, *Sc* Smooth clear, *Dmd* domed, *Ww* Watery white, *Rs* Raised, *W* White, *Cvx* Convex, − *ve* Gram negative, *T* Translucent, *O* Opaque, *Sg* Soft gummy, *C* Circular, *G* Gummy, *Fg* Firm gummy. Values within the nodulation column with common letters do not differ significantly according to Tukey’s HSD *p* < 0.05

### Molecular characterization of bradyrhizobia isolates

**Fig. 1:**
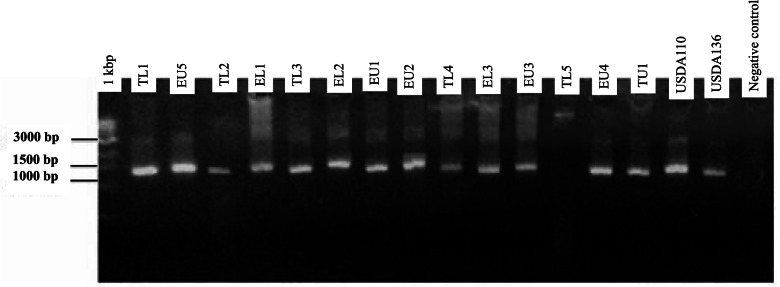
PCR amplification products of the 16S rDNA for representative bradyrhizobia isolates in 1.4% agarose gel

**Fig. 2:**
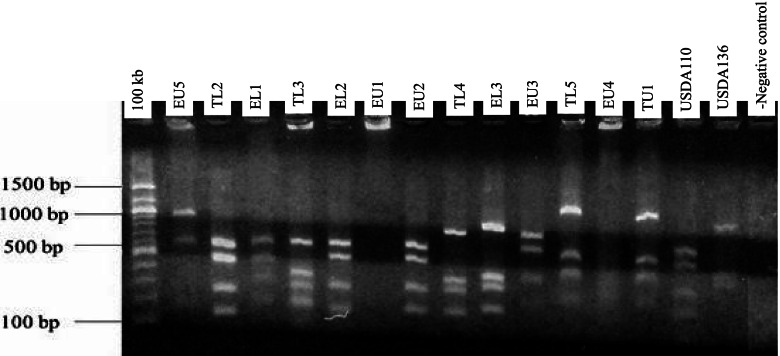
Restriction fragments for rhizobia isolates obtained after digestion with the *Msp*I enzyme in 1.4% agarose gel where DNA was stained with SYBR green

**Fig. 3:**
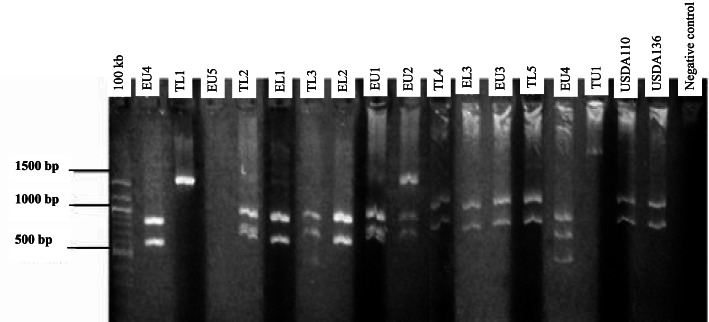
Restriction fragments for bradyrhizobia isolates obtained after digestion with the Eco RI enzyme on 1.4% agarose gel, and the DNA was stained with SYBR green dye

**Fig. 4:**
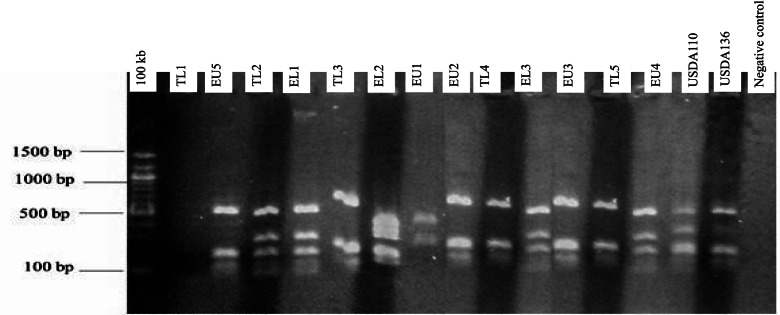
Restriction fragments for bradyrhizobia isolates obtained after digestion with the *Hae*III enzyme on 1.4% agarose gel

Genomic DNA was extracted from 38 indigenous bradyrhizobia isolates and two reference strains (USDA 110 and USDA 136). The PCR amplification of bradyrhizobia isolates and the two reference strains of soybeans produced a single band of 1500 base pairs in size using Y1 and Y3 primers (Fig. 1). Restriction digestion of 16S rRNA gene with enzymes *Hae*III, *Msp*I, and *EcoR*I resulted in different banding patterns. Digestion using *Msp*I enzyme resulted in fragments that ranged from 100 base pairs to 1000 base pairs (Fig. 2). The digestion with the *EcoR*I enzyme resulted in different restriction fragment patterns whose sizes ranged from 600 and 1200 base pairs (Fig. 3). Restriction digestion of 16S rRNA gene *Hae*III resulted to restriction fragments of sizes ranging from 100 base pairs to 600 base pairs (Fig. 4). The highest genetic variation (99%) was within populations (*p* = 0.015) and not among the regions or among the populations according to the analysis of molecular variance (AMOVA) (Table 4). The findings revealed the presence of diverse indigenuous bradyrhizobia isolates in the study sites that can possibly be utilized in the development of soybean biofertilizers.

**Table 4:** Analysis of molecular variance (AMOVA) for 38 bradyrhizobia isolates based on restriction digest of 16S rRNA using *Hae*III, *Msp*I, and *EcoR*I enzymes

Source	Df	Ss	MS	Est. Var	%	*P* value
Among regions	1	2.93	2.93	0.03	1	0.108
Among pops	2	4.70	2.35	0.00	0	0.815
Within pops	42	139.19	3.31	3.31	99	0.015
Total	45	146.83		3.35	100	

*Df* Degrees of freedom, *Ss* Sum of squares, *MS* Mean square, *Est*. *Var* Estimated variance, *Pops* Populations, *% Mol var* Percentage molecular variance

**Fig. 5:**
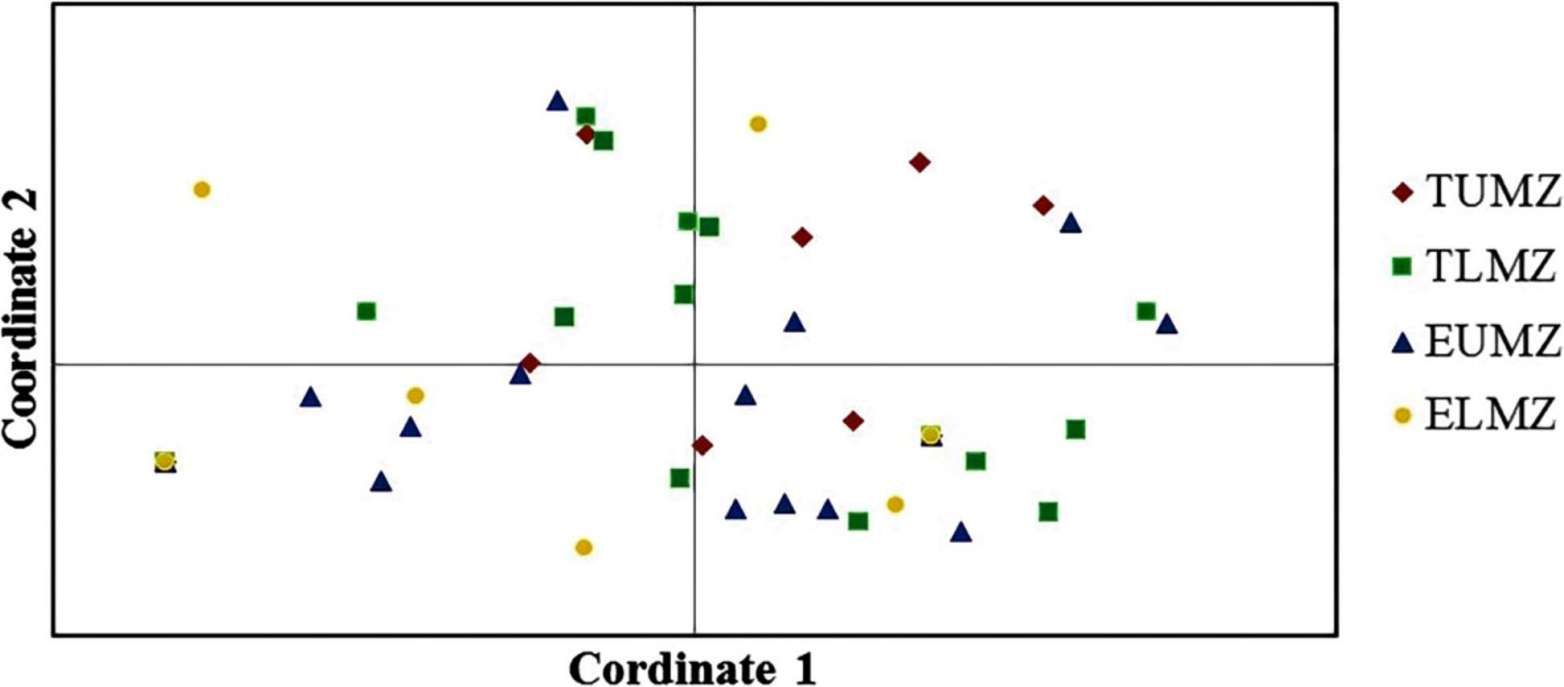
Principal coordinate analyses (PCA) of indigenous bradyrhizobia isolates based on restriction banding patterns. The percentage variations for the first two coordinates (1) 25.2% and (2) 17.6%

Principle coordinate analysis (PCA) of the 38 indigenous rhizobia isolates from the four agro-climatic zones showed some variations. Isolates were widely distributed in all the zones and overlapped in all the quadrants (Fig. 5). Isolates from EUMZ were the most widely distributed, appearing in all the quadrants while isolates from ELMZ were the least distributed (Fig. 5).

The highest genetic distance (0.039) based on pairwise Nei unbiased genetic assessment was between the population isolates from TUMZ and ELMZ. The lowest Nei unbiased genetic distance (0.012) was between rhizobia isolates populations from TUMZ and TLMZ and also between population isolates from TLMZ and EUMZ (Table 5).

**Table 5:** Nei unbiased genetic distance for pairwise population matrix of rhizobia populations trapped from the field in TUMZ, TLMZ, EUMZ, and ELMZ in Embu and Tharaka-Nithi Counties

TUMZ	TLMZ	EUMZ	ELMZ	
0.000				TUMZ
0.012	0.000			TLMZ
0.024	0.012	0.000		EUMZ
0.039	0.036	0.021	0.000	ELMZ

*TUMZ* Tharaka-Nithi Upper Midland Zone, *TLMZ* Tharaka-Nithi Lower Midland Zone, *EUMZ* Embu Upper Midland Zone, *ELMZ* Embu Lower Midland Zone

Indigenous bradyrhizobia populations from EUMZ and TUMZ had the highest mean number of alleles (Na = 1.6) compared to the other populations while ELMZ had bradyrhizobia populations with the lowest mean number of alleles (Table 6). The rhizobia populations were diverse according to the Shannon’s diversity index estimate (*H*′) where EUMZ and TLMZ showed the highest diversity estimates of *H’* = 0.419 and H = 0.404, respectively. The lowest genetic diversity (*H’* = 0.393) was recorded in isolates from ELMZ (Table 5). The highest percentage (80%) polymorphic loci (% P) was recorded from TUMZ and EUMZ bradyrhizobia populations while the populations from ELMZ had the lowest number of polymorphic loci (70%). The mean expected heterozygosity (He) for the indigenous bradyrhizobia populations ranged from 0.263 to 0.283 (Table 6). All the mentioned indicators suggest that bradyrhizobia populations from the ecological zones of eastern Kenya could be diverse.

**Table 6:** Mean number of different alleles (Na), number of effective alleles (Ne), Shannon’s Information Index (H′), expected heterozygosity (He), Unbiased expected heterozygosity (UHe), and percentage of polymorphic loci (% P) of indigenous rhizobia populations based on ARDRA data

Pop	Na	Ne	(*H*′)	He	UHe	%P
TUMZ	1.600 ± 0.18	1.437 ± 0.08	0.398 ± 0.057	0.263 ± 0.041	0.278 ± 0.044	80
TLMZ	1.500 ± 0.21	1.451 ± 0.08	0.404 ± 0.059	0.270 ± 0.041	0.279 ± 0.043	75
EUMZ	1.600 ± 0.18	1.496 ± 0.09	0.419 ± 0.061	0.283 ± 0.045	0.293 ± 0.046	80
ELMZ	1.400 ± 0.21	1.439 ± 0.08	0.393 ± 0.061	0.263 ± 0.042	0.284 ± 0.045	70

*TUMZ* Tharaka-Nithi Upper Midland Zone, *TLMZ* Tharaka-Nithi Lower Midland Zone, *EUMZ* Embu Upper Midland Zone, *ELMZ* Embu Lower Midland Zone

**Fig. 6:**
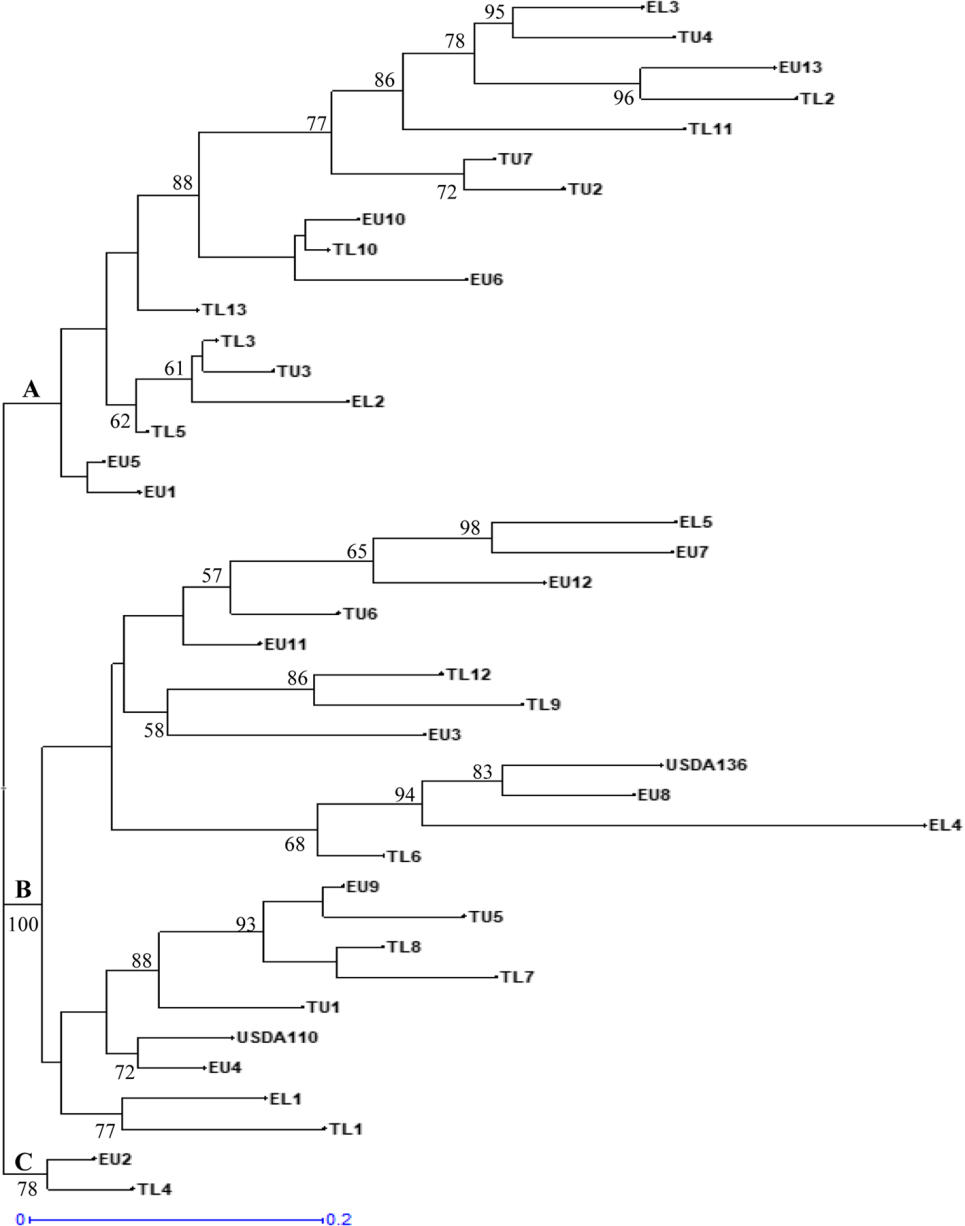
Phylogenetic relationship of 38 indigenous bradyrhizobia isolates and two reference strains (USDA110 and USDA136) based on combined *Hae*III, *Msp*I, and *EcoR*I restriction patterns of amplified 16S rRNA gene inferred using maximum likelihood method. Only bootstrap values ≥ 50% are shown

Phylogenetic analysis of the isolates inferred by maximum likelihood method using the three restriction enzymes showed distinct isolates. Indigenous bradyrhizobia isolates were clustered into three main clusters (clusters A, B, and C) (Fig. 6). Cluster A had the majority of the isolates with seventeen isolates that were distributed in different sub-clusters. Cluster B comprised of two main sub-clusters (Bi and Bii) with a bootstrap support of 100%. Bradyrhizobia isolate EU4 clustered together with the reference strain USDA110 while isolate EU8 clustered together with reference strain USDA136 showing they are closely related and supported by bootstrap values of 83% and 72%, respectively. Cluster C exclusively contained only two indigenous isolates (EU2 and TL4) supported by a bootstrap vale of 78% (Fig. 6).

**Fig. 7:**
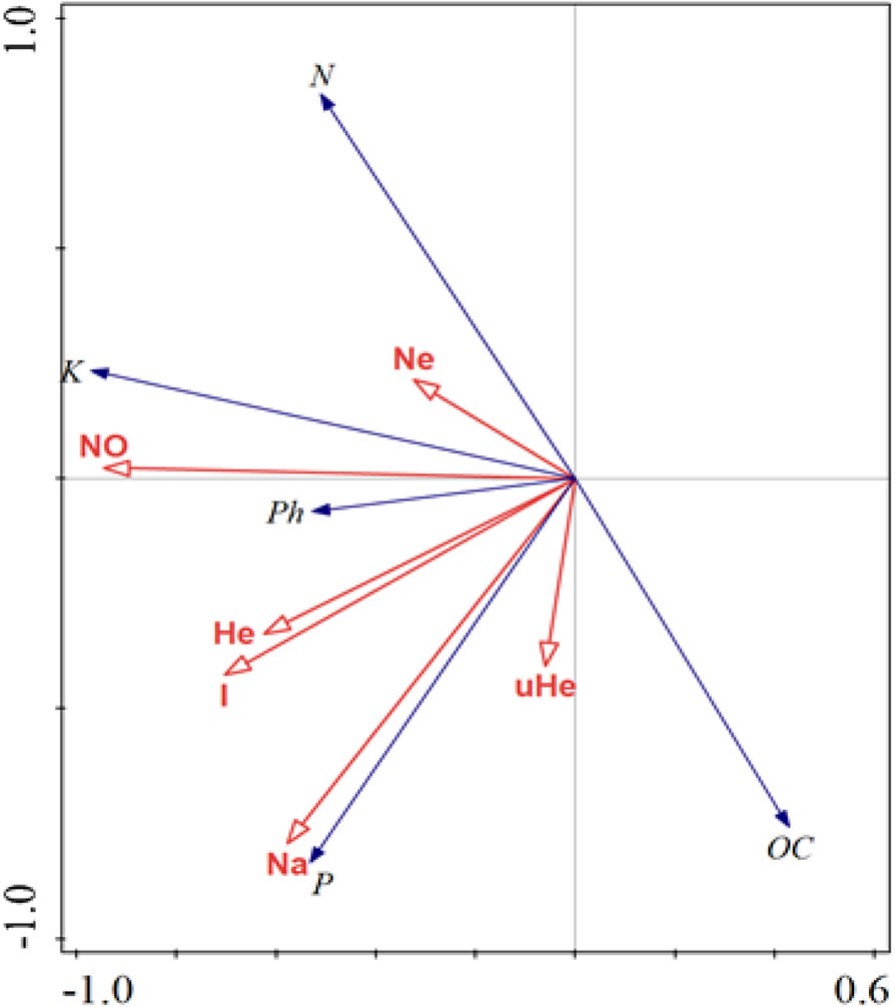
Canonical correspondence analysis (CCA) biplot analysis of bradyrhizobia isolates with soil physicochemical properties. OC, organic carbon; Ph, pH; K, potassium; P, available phosphorus; N, Total nitrogen; No, number of isolates; Na, number of alleles; Ne, number of effective alleles; I, information index; uHe, unbiased expected heterozygosity

Canonical correspondence analysis (CCA) suggests that environmental factors strongly correlated with microbial diversity (Fig. 7). The bacterial isolates and environmental factors canonical coefficients for the *x*-axis were higher than 0.6, while the *y*-axis was higher than 0.99. The length of the arrow indicated the relative importance of environmental factors in explaining the variation in microbial diversity observed. In addition, the angle between the diversity and microbial factors shows the degree of correlation. In this regard, OC, potassium (K), total nitrogen (N), and phosphorus (P) were the most important factors correlated with microbial diversity, as shown by their long arrows. The OC, pH, and P were strongly correlated with microbial diversity (I and uHe).

## Discussion

### Soil properties

The soils from the study sites were acidic while rhizobia require neutral pH to grow effectively. Legumes require neutral or slightly acidic soil with pH 6 or 7 for effective symbiotic association with rhizobia [43]. Although low soil pH is known to limit biological nitrogen fixation, some bradyrhizobia strains tolerate acidic soil and can compete effectively with other soil microorganisms and form nodules with host plants [55]. The soils from the study sites were deficient in phosphorus limiting nitrogen fixation; thus, farmers are left with the choice of using fertilizers to supplement the soil which is costly. Phosphorus functions as a source of energy in plants and hence influence the development of nodules. In legumes, phosphorus deficiency affects the development of root nodules, nitrogen acquisition, and nitrogen utilization [23]. According to Taliman et al. [50], phosphorus deficiency during soybean growth decreases nitrogen fixation.

### Bradyrhizobia morphological characteristics and symbiotic efficiency

There was a high diversity of bradyrhizobia isolates from the agroecological zones of eastern Kenya, as preliminarily suggested by the various colony characteristics of the isolates. The use of nodules trapped from the field allows for the isolation of diverse bradyrhizobia available in the soil [6]. In addition, the use of promiscuous soybeans which nodulate with diverse strains of bradyrhizobia as trap cultures permits the isolation of different isolates [8]. The isolates obtained had typical colony characteristics of bradyrhizobia as described by Somasegaran and Hoben [49]. Soybean-nodulating bradyrhizobia are slow growers (take up to 5 days) and alkali-producing in YEMA media supplemented with BTB [57]. Hannane et al. [14] while working with *Retama monosperma* (L.) Boiss similarly reported that bradyrhizobia are slow growers and alkaline producers hence BTB media take a characteristic blue color. Additionally, typical bradyrhizobia colonies when incubated in the dark showed little or no absorption of Congo Red [4]. Bradyrhizobia are differentiated from other bacteria such as *Agrobacterium* by non-absorption of Congo red in the dark [34]. However, some rhizobia according to Senthilkumar et al. [42] can absorb Congo red depending on the concentration of the dye, exposure to the light, and age of the culture producing deep pink or orange colonies. All the bradyrhizobia isolates obtained from the present study were gram-negative rods, circular, and with whitish colonies. This concurred with the findings by Kapembwa et al. [19] who reported 61 soybean-rhizobia isolates from three different agro-ecological regions of Zambia that morphologically varied with circular, cream, and white colonies being dominant.

Some isolates had larger colonies, which may be due to copious extracellular polysaccharides (EPS). Bradyrhizobia are known to produce surface polysaccharides (lipopolysaccharides and exopolysaccharides) that are believed to aid in stress adaptation and restrain the host’s defense mechanism [20]. In this study, isolates were obtained from areas with different agro-climatic conditions and production of polysaccharides may reflect their adaptation to the soils and climatic conditions of Embu and Tharaka-Nithi Counties. Boddey et al. [4] reported that bradyrhizobia isolates produced mucus as an adaptation to acidic soils. Mucus on bradyrhizobia has a crucial role in maintaining minimum moisture and preventing desiccation. Under nutrient-limiting conditions, they also act as a source of energy [19]. The in vitro mucus production indicates that the process and exo-polysaccharide composition entirely depend on the isolate genotype and not the host plant. This implies that bradyrhizobia isolates with the ability to produce mucus could have a higher competitive advantage in colonization and nodulation than non-mucus producers.

Inoculation of soybean varieties using native bradyrhizobia increase shoot dry weight and nodule dry weight significantly as compared to commercial inoculant Biofix and nitrogen treatment. The high nodulation after inoculation with specific isolates may be attributed to compatibility of the bacterial isolates with the promiscuous soybean varieties used [27]. To complete a substantive evaluation of the isolates, the isolates should be studied in terms of their competitive ability and agronomic effectiveness under field condition since the current assessment was only under axenic conditions without interaction with other soil microorganisms.

### Genetic diversity of indigenous bradyrhizobia isolates

Promiscuous soybeans are nodulated by diverse bacterial microsymbionts that can undergo genetic differentiation, leading to the emergence of new rhizobial types. The amplified 16S ribosomal DNA restriction analysis is an effective tool in assessing bradyrhizobial diversity due to restriction site variations of the genome in certain amplified regions [35]. In this study, the digestion of the 16S rRNA gene using restriction endonucleases differed among the different bradyrhizobial isolates based on the patterns, distance, and number of the digested fragments. The finding indicates that there is high variation among the indigenous isolates. This finding concurs with those reported in Mozambique where rep-PCR (BOX) and 16S rRNA sequencing revealed a remarkable diversity of soybean-bradyrhizobia native to the Mozambican agroclimatic conditions [8]. Despite the high bradyrhizobia diversity, the present study revealed that a few isolates exhibited close or similar fingerprints after restriction digest, indicating genetic relatedness. These findings are supported by the outcomes of Joglekar et al. [18], who reported bacterial isolates with similar banding patterns after digestion of ITS region using more than two endonucleases.

Based on the analysis of molecular variance (AMOVA), most of the genetic variation of the isolates was within populations. There was a significantly (*p* < 0.05) high level of variation partitioned within bradyrhizobia populations, confirming diverse local soybean microsymbionts. According to Riah et al. [39], this suggests that there are limited physical barriers to the flow of the gene and rhizobia populations from the study zones are not well structured. These findings concur with those of Shiro et al. [46] who recorded a high genetic variation of bradyrhizobia isolates within populations isolated from different agro-climatic conditions in the northern and southern USA. Similarly, in this study, PCA analysis of the bradyrhizobia isolates showed adequate genetic variation within populations, and notably, majority of the bradyrhizobia isolates were present in all the agroecological zones as indicated in the clustering. Jaiswal et al. [17] documented that PCA analysis of cowpea bradyrhizobia isolates was independent of the regions they were isolated, which support the findings from the present study. The high diversity within the agroecological zones may be due to the high nitrogen demand by the crop, which can stimulate nodule formation leading to bradyrhizobia proliferation [15]. Different soil properties and land use practices can also lead to variations in bradyrhizobia diversity [34].

The correspondence analysis indicated that soil organic compound, available P, and total N were the important factors influencing soil microbial diversity. According Mburu et al. [26], plant growth promoting rhizobacteria (PGPR) mediate key soil processes involved in nitrogen, phosphorus and carbon cycling, which potentially determine correlation between environmental factors and microbial structure. Growth limiting resources available in the soil such as organic compounds in detritus influence microbial diversity by generating cellular energy, and especially for heterotrophic bacterial communities [59]. Besides, total N saturation in the soil can lower soil pH leading to mobilization of aluminum ions that cause aluminum toxicity [10]. Therefore, soil microbial diversity is highly influenced by environmental factors as demonstrated in this study.

The genetic relatedness of the indigenous bradyrhizobia isolates based on ARDRA analyses of the 16S rDNA showed a narrow Nei unbiased genetic distance. The use of soybeans as the trap cultures could contribute to this observation where only specific strains of bradyrhizobia can effectively nodulate [30]. In addition, this observation could also be due to the targeted 16S rRNA gene which might be affected by horizontal gene transfer and genetic recombination among the soil bradyrhizobia [30, 40]. Ferraz-Helene et al. [12] reported similar findings where bradyrhizobia populations isolated from different agroecological zones in South Africa and Western Australia had a close genetic distance of housekeeping genes (*dnaK*, *glnII*, *gyrB*, *recA*).

A dendrogram constructed from the 16S rRNA digestion profiles clustered the bradyrhizobia isolates into different groups. This variability of bacterial isolates confirms the presence of diverse soybean bradyrhizobial isolates in the local microsymbiont population. The diversity may be contributed by trapping the isolates from their natural environment and by using promiscuous soybeans that form a symbiosis with diverse strains of bradyrhizobia [6, 8]. Similar findings have been reported for indigenous rhizobia in *Phaseolus vulgaris* L. from various agroecological zones in Nepal [40]. Interestingly, the two isolates from this study that clustered together with the reference strains were obtained from the same agroecological zone in EUMZ. Most of the indigenous bradyrhizobia isolates did not cluster together with the reference strains USDA 110 and USDA 136 showing that they were distinct. The three main clusters of soybean bradyrhizobia isolates were distributed into distinct sub-clusters that depict the isolates’ extensive diverse heterogeneity from Eastern Kenya. This demonstrates that sufficient gene diversity exist among the isolates. Similarly, Koskey et al. [21] studied 41 climbing bean-rhizobia isolates using three restriction endonucleases and reported a significant (*p* < 0.05) genetic variation based on PCR-ARDRA profiling. The high isolate diversity in the soil increases the chance for any legume crop to find compatible rhizobia effective in biological nitrogen fixation.

## Conclusion

In this study, 38 bradyrhizobial isolates were obtained from the soybean nodules and they exhibited varying phenotypic and genotypic characteristics. This underscores that microsymbionts isolated from promiscuous soybeans in Eastern Kenya consist of different bradyrhizobia. Some of the indigenous isolates exhibited superior symbiotic traits and they are being considered for further assessment under field conditions. Analysis of molecular variance revealed significant (*p* < 0.05) genetic variation within populations, showing that the region is a hub for diverse bradyrhizobia isolates. Cluster analyses based on molecular characteristics revealed a high level of genetic diversity between the soybean bradyrhizobia isolates and the two reference strains, indicating that the soils from the study sites harbor diverse strains of bradyrhizobia*.* The canonical correspondence analysis revealed the importance of organic carbon, pH, and phosphorus in correlation with bacterial diversity from different agroecological zones. Therefore, this study creates a vital ecological framework that can be applied in developing effective microsymbionts that are competitive, adapted to the environment, and have greater nitrogen-fixing ability. The phylogenetic classification and precise taxonomy of the bradyrhizobia strains isolated in the present study should be further studied to detail their diversity and identity. 

## Data Availability

The datasets used in this study are available upon request through the corresponding author.

## Abbreviations

ARDRA: Amplified ribosomal DNA restriction analysis
AMOVA: Analysis of molecular variance
PCR: Polymerase chain reaction
YEMA: Yeast extract mannitol agar
TBE buffer: Tris-borate-EDTA buffer
TUMZ: Tharaka-Nithi Upper Midland Zone
TLMZ: Tharaka-Nithi Lower Midland Zone
EUMZ: Embu Upper Midland Zone
ELMZ: Embu Lower Midland Zone
